# Immediate vs delayed left bundle branch block after transcatheter aortic valve replacement: incidence and risk factors for permanent pacemaker implantation

**DOI:** 10.1007/s10840-026-02248-3

**Published:** 2026-02-19

**Authors:** Maayan Shrem, Oholi Tovia-Brodie, Moshe Rav Acha, Ziv Dadon, Maxime Zabern, Nimrod Perel, Danny Dvir, Michael Glikson, Yoav Michowitz

**Affiliations:** 1 Cardiology Department, Jesselson Integrated Heart Center and Eisenberg R&D Authority, Shaare Zedek Medical Center, Jerusalem, Israel; 2 Faculty of Medicine, Hebrew University, Jerusalem, Israel; 3 Division of Cardiovascular Medicine, Cardiac Electrophysiology Section, Shamir Medical Center, Be’er Yaakov, Israel

**Keywords:** Left Bunder Branch Block, Transcatheter aortic valve replacement (TAVR), Permanent pacemaker implantation (PPI), Delayed, Immediate

## Abstract

**Background:**

Characteristics of patients with delayed compared to immediate new onset left bundle branch block (LBBB) after transcatheter aortic valve replacement (TAVR) are poorly defined.

**Objectives:**

To describe the incidence and features of patients with delayed post TAVR LBBB as well as risk factors for permanent pacemaker implantation (PPI).

**Methods:**

1469 consecutive TAVR patients were screened for the occurrence of new onset LBBB. ECG was performed within 1 week before the procedure, immediately post procedure, and daily thereafter until discharge. According to the immediate post TAVR ECG, patients were divided into 2 groups, immediate and delayed new onset LBBB. Both groups were followed for the occurrence of PPI and mortality.

**Results:**

Among patients who underwent TAVR, 296 (20.1%) developed new-onset LBBB, of whom, 282 were included the present study. LBBB occurred immediately after the procedure in 249 patients (88.3%) and was delayed in 33 patients (11.7%). Rates of both early PPI within 2 weeks as well as long term PPI and mortality were comparable between patients with immediate vs. delayed onset LBBB. In patients with immediate LBBB, QRS width correlated with a need for PPI, while in patients with delayed LBBB, PR prolongation of ≥ 115 ms differentiated between patients with and without PPI.

**Conclusions:**

The appearance of new onset post TAVR LBBB may be delayed in ~ 12% of patients. These patients exhibit similar rates of PPI need and mortality; however different ECG parameters signify high risk for PPI need among these patients.

**Graphical Abstract:**

**Figure:**
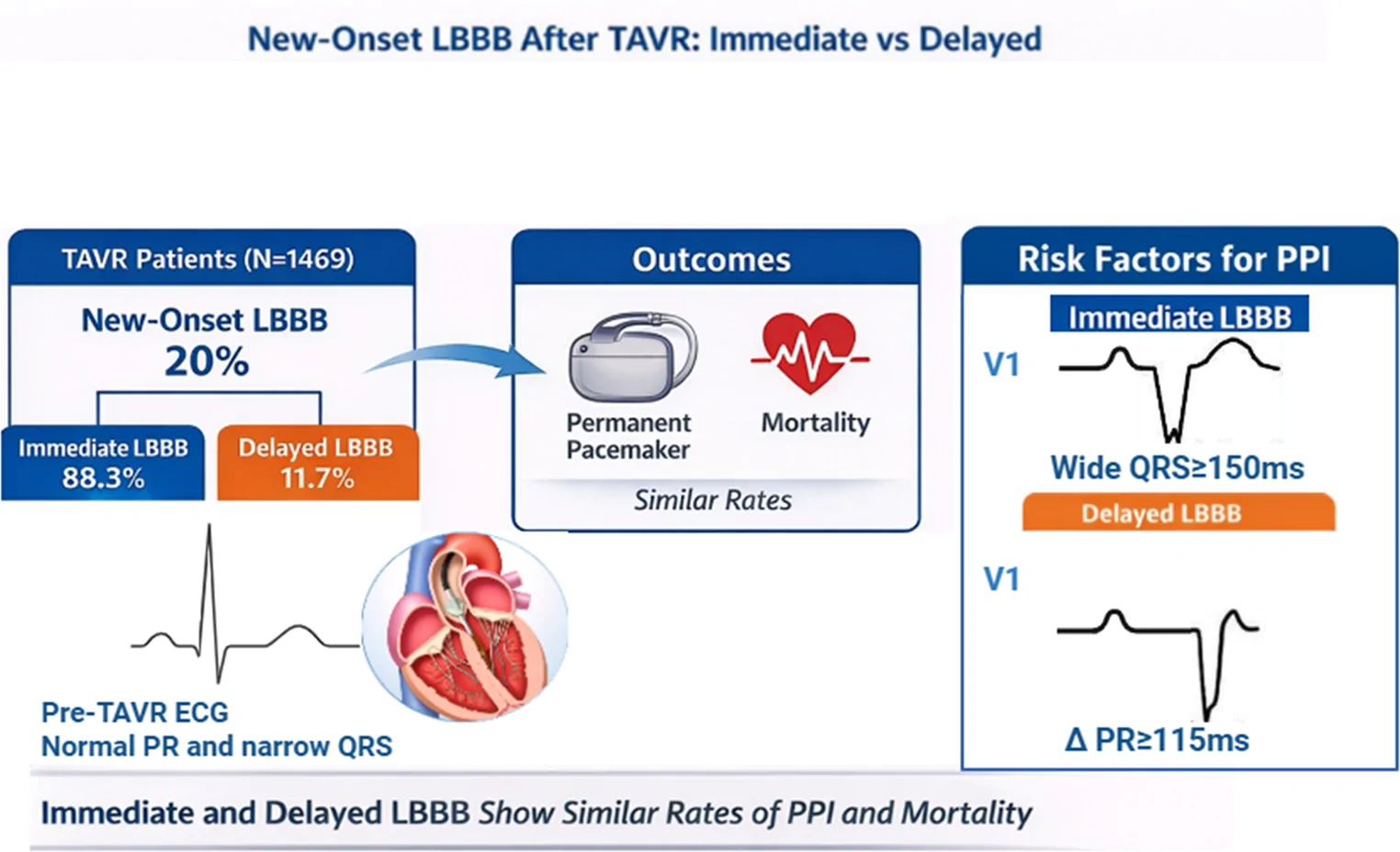

**Supplementary Information:**

The online version contains supplementary material available at https://doi.org/10.1007/s10840-026-02248-3.

## Introduction

Left bundle branch block (LBBB) is the most common conduction abnormality occurring after transcatheter aortic valve replacement (TAVR) [1–5]. Multiple studies have examined its predictive value for the development of advanced atrioventricular block (AVB) and need for permanent pacemaker implantation (PPI), as well as its long-term effect on left ventricular function and patients’ overall survival [6, 7].

Most commonly, new-onset TAVR related LBBB occurs during the procedure and is recorded immediately on the first post-TAVR electrocardiogram (ECG) [8, 9]. Nevertheless, few studies have shown that the appearance of post-TAVR new-onset LBBB may be delayed. Houthuizen et al. reported 12% incidence of subacute LBBB [10], defined as occurring more than 24 h after TAVR, while Urena et al. described an incidence of 6.5%, with delayed onset LBBB occurring in 4 out 61 cases of new-onset post-TAVR LBBB [6].

The above-mentioned studies focus was on the persistence of LBBB during hospitalization and during follow-up and its relation to prognosis [6, 7, 10]. Other studies demonstrated that post-TAVR conduction abnormalities are dynamic and may progress or regress during the first post procedural days [1, 11, 12]. Nevertheless, the characteristics and clinical significance of immediate versus delayed post TAVR new-onset LBBB have not been studied. As different mechanisms may underline the development of immediate versus delayed post TAVR LBBB, it seems plausible that their clinical characteristics, course, and optimal management may differ. Accordingly, this study aimed to characterize delayed new-onset LBBB following TAVR and determine whether it has distinct features.

## Methods

### Patient population

This study included patients who underwent TAVR between 2010 and 2022 and had new-onset LBBB documented on ECG. Patients were excluded if they had: (i) pre-existing bundle branch block (BBB) prior to TAVR; (ii) a history of prior TAVR or surgical aortic valve replacement (SAVR); (iii) a pre-existing permanent pacemaker; (iv) missing documentation of LBBB post-TAVR; or (v) newly developed right bundle branch block (RBBB) after the procedure.

Clinical, procedural, electrocardiographic, and echocardiographic data were obtained through manual review of electronic medical records. The study protocol was approved by the institutional ethics committees, and the requirement for written informed consent was waived due to the retrospective nature of the analysis and the use of deidentified data.

Collected baseline and post-procedure ECG parameters included: rhythm, electrical axis, PR interval, QRS morphology and duration. Post-TAVR ECGs were reviewed from the time of post procedure admission to the cardiac intensive care unit or cardiology department (first post-TAVR ECG) and daily thereafter until discharge. Patients were included if they had documented new-onset LBBB at any point during the index hospitalization. They were then stratified into two groups based on the timing of LBBB onset: **immediate LBBB** was defined as the documentation of a new post-TAVR LBBB on the first post-TAVR ECG, and **delayed LBBB**, when the first post-TAVR ECG demonstrated narrow QRS and LBBB was documented on subsequent ECGs during hospitalization.

The incidence of in hospital AVB, alternating BBB, performance of electro-physiological study (EPS) and the need for PPI were recorded. Definitions of BBB and fascicular blocks followed the American Heart Association/American College of Cardiology Foundation/Heart Rhythm Society recommendations [13] and the 2021 European Society of Cardiology guidelines on cardiac pacing and resynchronization therapy [3].

### Follow-up

Patients were followed for the need of PPI, including clinical indications, as well as all-cause mortality. For the analysis of PPI as an adverse event, only patients with a relevant clinical indication were included. These indications comprised complete or high-degree AVB, alternating BBB, or a PPI indication established by EPS [3]. Device implantation due to sick sinus syndrome (SSS), ventricular arrhythmias, or heart failure (i.e., implantable cardioverter defibrillator or cardiac resynchronization therapy implantation) were not considered TAVR related PPI. Follow-up was conducted via electronic health records and phone call when needed.

### Statistical analysis

Continuous variables were assessed for distribution using histograms and are reported as means with standard deviations. Categorical variables are presented as frequencies and percentages. Comparisons between groups (immediate and delayed LBBB post-TAVR) were made using the Chi-square or Fisher’s exact test for categorical variables, and the Mann–Whitney U test for continuous variables.

Kaplan–Meier survival analyses were performed to estimate the cumulative incidence of all-cause mortality and PPI and log rank test was used to compare between the various groups. The area under the receiver-operating characteristic (ROC) curve was used to evaluate the discriminating ability of maximal Δ PR and maximal QRS on the occurrence of PPI in patients with immediate and delayed post-TAVR LBBB. Maximal Youden index was applied to identify the optimal cutoff value. Multivariable logistic regression was used to identify independent predictors that are associate with delayed LBBB and with PPI within 2 weeks. Clinically relevant variables with *p* < 0.1 were entered into the regression using backwards elimination methods (*p* > 0.1 in Wald test was used as a criterion for variable removal). In the next block, age and sex were forced into the regression. Since, in patients with delayed post TAVR LBBB, the absolute number of PPI events was < 10, multivariable analysis was not applied for this group. A two-sided *p*-value < 0.05 was considered statistically significant. All statistical analyses were conducted using IBM SPSS Statistics, version 29.0.2.0 (Chicago, IL, USA).

## Results

### Study population

Between May 2010 and December 2022, a total of 1469 TAVR procedures were performed at our institution, among which, 107 patients already had a permanent pacemaker in place and 62 had baseline BBB before the TAVR. Three hundred and nine (21%) patients developed new BBB following the procedure. Of these, the following were excluded; 13 developed RBBB [5], 7 had previous TAVR/ SAVR and underwent valve in valve procedure and 7 underwent TAVR for aortic insufficiency. Therefore, 282 patients were eligible for analysis in the current study. These were categorized based on the timing of new-onset LBBB detection: 249 patients (88.3%) as the immediate LBBB group and 33 (11.7%) as the delayed LBBB group. Of note, 1 (3%) patient from the delayed-LBBB group had axis change on his first post TAVR ECG before developing LBBB. Median follow-up was 3.4 years (IQR 2.1–5.2 years).

As detailed in Table 1, baseline clinical characteristics were comparable between patients with delayed versus immediate post TAVR LBBB. However, few procedural details were different; valve size was marginally larger (*p* = 0.058) and use of temporary pacemaker was lower in patients with delayed-onset post-TAVR LBBB (*p* < 0.001). Use of balloon-expandable vs. self-expandable valves were similar (*p* = 0.55). Eight (24%) patients from the delayed LBBB group had transient QRS widening during the TAVR, however their first post TAVR ECG demonstrated narrow QRS. Post procedurally, EPS was performed in 6 (18.2%) and 29 (11.6%) patients from the immediate and delayed LBBB groups, respectively (*p* = 0.27). Atrio-His (AH) interval was non-significantly longer in the delayed LBBB group (184.7 ± 39.8 ms vs. 153.9 ± 55.4 ms, *p* = 0.21) and His-ventricular interval (HV) was similar (62.8 ± 15.7 ms vs. 63.5 ± 9.9 ms, *p* = 0.89), respectively. Supplementary Table 1. Rates of in hospital, AVB, PPI and death were comparable between the 2 groups, however, hospitalization duration was longer in patients with delayed post TAVR LBBB [7.7 days (IQR 3–9) versus 6.2 days (IQR 3–6), respectively, *p* = 0.014].

**Table 1:** Baseline clinical characteristics

	Immediate LBBB	Delayed LBBB	*p* Value
	(*n* = 249)	(*n* = 33)	
Age (years)	80.58 ± 7.2	80.76 ± 7.9	0.656
Female sex (%)	125 (50.2)	18 (54.5)	0.639
BMI (Kg/m^2^)	27.7 ± 5.1	29.5 ± 5.1	0.052
Diabetes Mellitus (%)	103 (41.4)	15 (45.5)	0.655
Hypertension (%)	214 (85.9)	29 (87.9)	> 0.99
Hyperlipidemia (%)	178 (71.5)	27 (81.8)	0.211
Chronic Renal Failure (%)	55 (22.1)	4 (12.2)	0.186
Atrial Fib/Flutter (%)	65 (26.1)	13 (39.4)	0.109
Prior IHD (%)	128 (51.4)	16 (48.5)	0.752
Beta-blockers (%)	143 (57.4)	23 (69.7)	0.178
ACE inhibitors/ARB (%)	150 (60.2)	18 (54.5)	0.531
Amiodarone (%)	31 (12.4)	4 (12.1)	> 0.99
LVEF (%)	55.6 ± 7.9	57.1 ± 6.1	0.371
Aortic valve area (cm^2^)	0.63 ± 0.15	0.59 ± 0.14	0.122
Peak gradient (mmHg)	81.5 ± 25.2	81.3 ± 26.4	0.977
Mean gradient (mmHg)	48.2 ± 15.5	48.7 ± 17.1	0.907
Calcium score (HU)^	2568 ± 1674.9	2467 ± 1435.2	0.898
Procedural characteristics			
Trans femoral access	247 (99.2)	33 (100.0)	> 0.99
Valve mechanism (BEV/SEV)	107(43.0)/142(57.0)	16(48.5)/17(51.5)	0.548
Valve size	26.4 ± 2.4	27.4 ± 2.9	0.058
Transient CAVB during TAVR	14 (5.6)	1 (3.0)	> 0.99
Transient QRS widening during TAVR	0 (0)	8 (24.2)	< 0.001
Temporary pacemaker	185 (74.3)	10 (30.3)*	< 0.001
In hospital post procedure events/ management			
EPS	29 (11.6)	6 (18.2)	0.269
CAVB or HDAVB during hospitalization	22 (8.8)	5 (15.2)	0.347
In hospital PPI	31 (12.4)	7 (21.2)	0.176
Days to in hospital PPI	3 (1–6)	3 (2–6)	0.97
Death in hospital	2 (0.8)	2 (6.1)	0.069
Hospitalization time (days), median (IQR)	6.2 (3–6)	7.7 (3–9)	0.014

*AADs* Anti arrhythmic drugs; *ARB* Angiotensin II receptor blocker; *ACE* Angiotensin converting enzyme; *BEV* Balloon-expandable valve; *BMI* Body mass index; *CAVB* Complete atrioventricular block; *EPS* Electrophysiological study; *HDAVB* High-degree atrioventricular block; *IHD* Heart ischemic disease; *LVEF* Left ventricular ejection fraction; *PPI* Permanent pacemaker implantation; *SEV* Self-expandable valve; *TAVR* Transcatheter aortic valve replacement

^*^ 7 patients were left with temporary pacemaker immediately post TAVR due to: transient AVB (*n* = 1), transient QRS widening (*n* = 4), cardio pulmonary resuscitation during TAVR (*n* = 1), and junctional rhythm during TAVR (*n* = 1). Another 3 received temporary pacemaker post procedure due to development of delayed post TAVR LBBB

^Data not available for 75 and 8 patients from the immediate and delayed LBBB subgroups, respectively

ECGs were available for all 282 patients, including baseline, daily post-procedural recordings, and ECGs performed prior to hospital discharge.

Table 2 outlines the pre- and post-TAVR ECG parameters of the 2 patients’ groups. A significant difference was observed in pre-TAVR QRS axis distribution, with a higher proportion of patients in the immediate LBBB group exhibiting a normal axis compared to the delayed LBBB group [66.7% vs. 54.5%, respectively; *p* = 0.044]. The immediate LBBB group also demonstrated a trend toward longer pre-TAVR PR intervals compared to the delayed group [188.9 (IQR 160-212ms) vs. 174.1 (IQR 154-188ms); *p* = 0.056].

**Table 2:** Pre and post TAVR ECG parameters of patients with immediate and delayed LBBB

	Immediate LBBB	Delayed LBBB	*p* Value
	(*n* = 249)	(*n* = 33)	
Pre TAVR ECG			
Sinus rhythm	228 (91.6)	27 (81.8)	0.106
QRS axis (N/L/R)	166 (66.7)/83 (33.3)/0 (0.0)	18 (54.5)/ 14 (42.4)/ 1(3.0)	0.044
PR duration* (ms)	188.9 ± 38.6	174.1 ± 27.3	0.056
QRS duration (ms)	92.8 ± 12.7	92.9 ± 11.8	0.811
Post TAVR ECG			
Sinus rhythm	227 (91.2)	27 (81.8)	0.115
QRS axis (N/L/R)	57 (22.9)/ 189 (75.9)/ 3 (1.2)	5 (15.2)/ 28 (84.8)/ 0 (0.0)	0.571
Axis change (%)	120 (48.2)	14 (42.4)	0.533
Post TAVR PR duration (ms)^*,$^	202.9 ± 37.6	200.4 ± 49.9	0.427
Post TAVR QRS duration (ms)^$^	145.3 ± 15.3	142.2 ± 14	0.329
Maximal PR duration (ms)*^,#^	227.68 ± 42.7	239.5 ± 45.6	0.16
Maximal QRS duration (ms)^#^	149.9 ± 16.2	146.8 ± 16.2	0.392
Δ PR (ms)*	14.3 ± 34.5	23.8 ± 46.1	0.829
Maximal Δ PR* (ms)^&^	39.1 ± 34.4	65.5 ± 48.3	0.005
Δ QRS (ms)	52.4 ± 16.5	49.3 ± 15.3	0.276
Maximal Δ QRS (ms)^&^	57.06 ± 16.6	53.8 ± 15.9	0.292
Days to LBBB	0	1.88 (1–2.5)	< 0.001
Day of maximal PR duration*	1.63 (0–3)	3.54 (2–4.75)	< 0.001
Day of maximal QRS duration	0.88 (0–1)	2.58 (1–3.5)	< 0.001
Persistent LBBB upon discharge^	142 (57)	24 (72.7)	0.09

*LBBB* Left bundle branch block; *N/L/R* Normal/left/right; *PPI* Permanent pacemaker implantation; *TAVR* Transcatheter aortic valve replacement

^*^ Data not available for 25 patients with atrial fibrillation (20 with immediate LBBB and 5 with delayed LBBB)

^$^ In first ECG documentation of LBBB after TAVR

^#^ Longest documented PR/QRS during hospital follow-up

^&^ Δ between maximal PR/QRS to pre-TAVR PR/QRS

^ Persistent to discharge or to in hospital pacemaker implantation or mortality

As per inclusion criteria (new-onset LBBB), QRS duration increased post-procedurally, from 92.8ms (IQR 82–101ms) to 145.3ms (IQR 134–155ms) in the immediate LBBB group and from 92.9ms (IQR 84–100ms) to 142.2ms (IQR 132–157ms) in the delayed LBBB group (*p* = 0.329). In addition, as expected, the median day of LBBB onset differed significantly between the groups, occurring on day 0 (same day as the TAVR) in the immediate group and on day 1.88 (IQR 1–2.5) in the delayed group (*p* < 0.001).

The timing of maximal PR prolongation and QRS widening also differed, with earlier peaks in the immediate LBBB group compared to the delayed group: 1.63 (IQR 0–3) vs. 3.54 (IQR 2–4.75) days for PR interval and 0.88 (IQR 0–1) vs. 2.58 (IQR 1–2.5) days for QRS duration, respectively (*p* < 0.001 for both). The change in PR interval from baseline to maximum post-TAVR was significantly greater in the delayed LBBB group (65.5ms ± 48.3 vs. 39.1 ± 34.4ms; *p* = 0.005). Other ECG parameters did not differ significantly between the two groups.

### Multivariate analysis for the occurrence of delayed-onset LBBB

Multivariable logistic regression was used to identify independent predictors that are associate with post-TAVR delayed onset LBBB (based on univariate analysis from Tables 1 and 2). The model included 4 variables: BMI, valve size, baseline QRS axis and baseline PR interval. Valve size (OR 0.75, 95% CI 0.62–0.89, *p* = 0.001) and PR interval (OR 1.02, 95% CI 1.002–1.03, *p* = 0.026) were independently associated with delayed onset LBBB. The association remained statistically significant even after adjusting for age and sex.

### PPI and mortality during follow-up

**Fig. 1:**
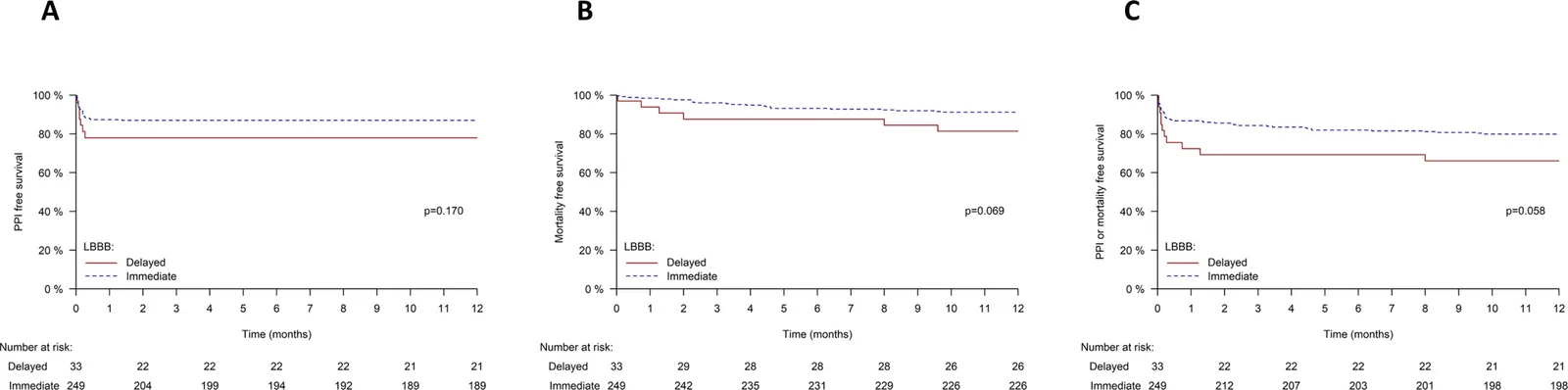
Kaplan–Meier curves for **A**-permanent pacemaker implantation, **B**-Mortality, **C**-combined endpoint of PPI and mortality during 1-year follow-up. A nonsignificant worst outcome is shown in all curves for patients with delayed (red line) compared to immediate (dotted blue line) post TAVR LBBB

During the first-year post TAVR, 32 (12.8%) and 7 (21%) patients underwent PPI (*p* = 0.19) due to relevant indication from the immediate and delayed post-TAVR LBBB groups, respectively. All these PPIs occurred during the first 2 weeks post-TAVR except for 1 PPI from the immediate LBBB group that occurred 41 days post-TAVR. PPI indications during the first 2-weeks post TAVR, included AVB in 24 (77.4%) vs 5 (71.4%), alternating BBB in 3 (9.7%) vs 1 (14.3%) and EPS demonstrating prolonged HV > 70 ms in 4 (12.9%) vs 1 (14.3%), *p* = 0.93; in the immediate and delayed LBBB subgroups, respectively. Mortality events during this period of one year, occurred in 50 (20%) and 11 (33.3%), *p* = 0.11, from the immediate and delayed post TAVR LBBB groups, respectively. KM curves of 1- and 5-years follow-up, for PPI, mortality and the combined endpoint of PPI and mortality are presented in Fig. 1 and Supplementary Fig. 1, respectively. Patients in the delayed LBBB group had a nonsignificant worse outcomes during 1- and 5-years follow-up of both need for PPI and mortality.

### Predictors for PPI in patients with immediate vs. delayed post TAVR LBBB

**Fig. 2:**
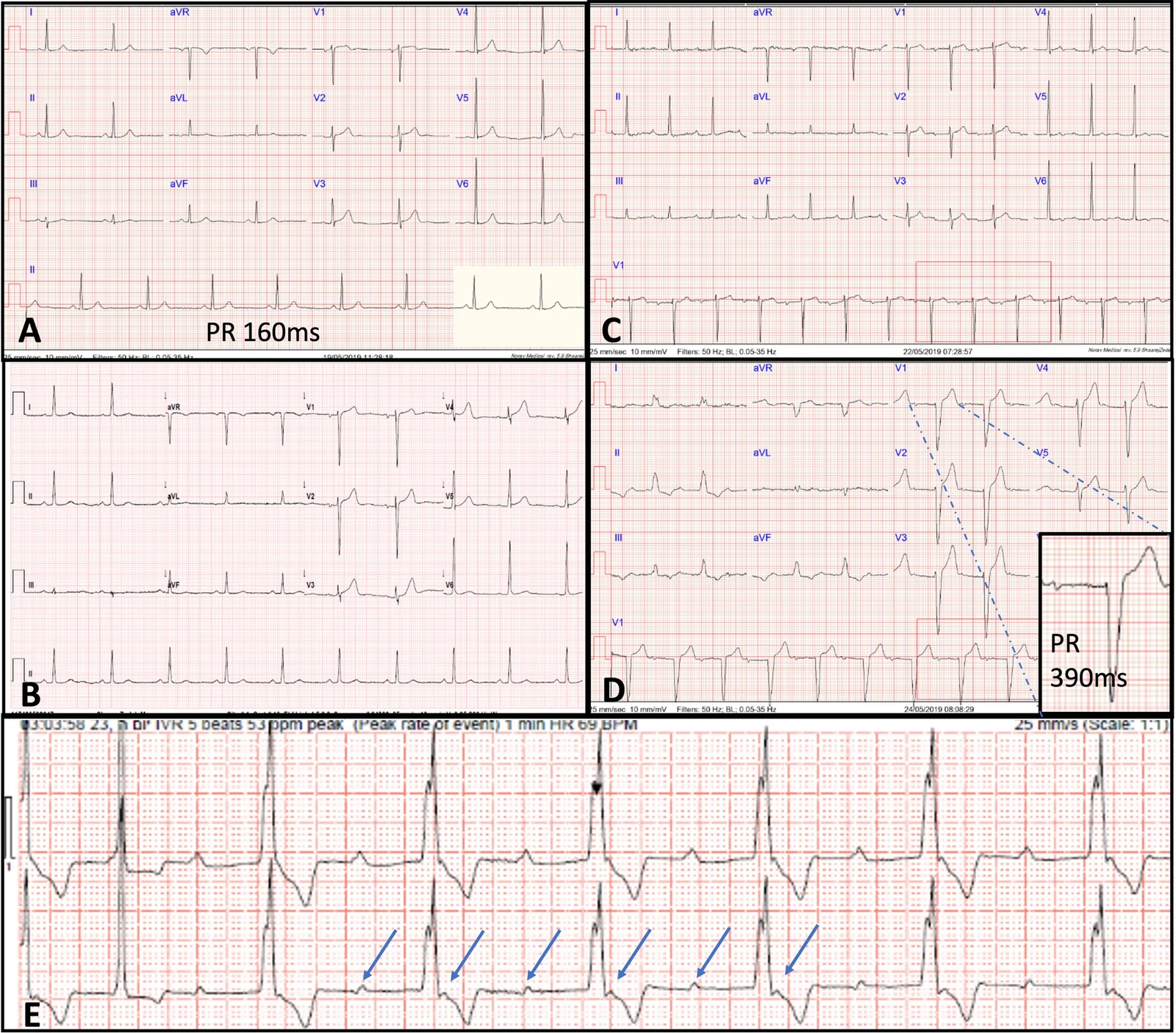
Serial ECGs of an exemplary patient with delayed LBBB. **A** – pre-TAVR baseline ECG, PR 160ms and narrow QRS are shown. **B** – Immediate post-TAVR ECG showing PR 190ms with narrow QRS. **C** – ECG post TAVR showing PR prolongation to 300ms with narrow QRS. **D** – First documented LBBB post-TAVR, PR 390ms (ΔPR = 230ms) and QRS 135ms with LBBB morphology. **E**—24h Holter monitoring demonstrating 2:1 AVB, blue arrows point to P waves

Table 3 and Supplementary Table 2 demonstrate separate comparisons for patients with immediate and delayed post-TAVR LBBB. In each patients’ group, a comparison is made between those with and without PPI within 2-weeks post-TAVR. In patients with immediate -onset LBBB, baseline, post procedural and maximal QRS width were significantly wider in patients who underwent PPI compared to those who did not. Maximal Δ PR was marginally longer in patients with PPI (*p* = 0.052). In addition, persistence of LBBB was more common among patients who underwent PPI. In contrast, in patients with delayed-onset LBBB, neither QRS width (baseline, post procedural and maximal QRS width), nor LBBB persistence to discharge, were significantly different between patients with and without PPI. Rather, baseline PR was shorter and maximal Δ PR was longer in patients with PPI compared to those without PPI. Of note, among patients with delayed-onset LBBB, PPI was more common among men compared to women. An example of sequential ECG changes in a patient with post-TAVR delayed-onset LBBB is presented in Fig. 2.

**Table 3:** ECG parameters of patients with immediate and delayed LBBB with and without early PPI within 2 weeks

	Immediate LBBB		*p* Value	Delayed LBBB		*p* Value
	(*n* = 247) *			(*n* = 32) *		
	No PPI (*n* = 216)	PPI (*n* = 31)		No PPI (*n* = 25)	PPI (*n* = 7)	
Pre TAVR ECG						
Sinus Rhythm	199 (92.1)	27 (87.1)	0.313	21 (84.0)	5 (71.4)	0.59
QRS axis (N/L/R)	150(69.4)/66(30.6)/0(0)	15(48.4)/16(51.6)/0(0)	0.02	14 (56)/11(44)/0(0)	3(42.9)/3(42.9)/1(14.3)	0.292
PR duration^**^ (ms)	189.1 ± 38.9	189.6 ± 37.6	0.613	180.8 ± 26.7	150.8 ± 14.3	0.019
QRS duration (ms)	92.31 ± 12.8	97.16 ± 11.3	0.048	94.32 ± 11.9	89.43 ± 11.5	0.324
Post TAVR ECG						
Sinus rhythm	197 (91.2)	28 (90.3)	0.745	21 (84.0)	5 (71.4)	0.59
QRS axis (N/L/R)	51(23.6)/163(75.5)/2(0.9)	6(19.4)/25(80.6)/0(0.0)	0.196	3(12)/22(88)/0(0)	2(28.6)/5(71.4)/0(0)	0.296
Axis change (%)	105 (48.6)	14 (45.2)	0.719	11 (44)	2 (28.6)	0.671
Post TAVR PR duration (ms)^**,$^	201.7 ± 37.4	212.2 ± 39.6	0.174	197.8 ± 43.7	219.2 ± 76.0	0.569
Post TAVR QRS duration (ms)^$^	144.5 ± 15.2	150.9 ± 15.0	0.021	141.3 ± 14.3	143.2 ± 13.0	0.688
Maximal PR duration (ms)^**,#^	226.4 ± 43.3	238.4 ± 36.2	0.136	235.9 ± 46.1	265.0 ± 26.8	0.195
Maximal QRS duration (ms)^#^	148.7 ± 15.8	158.2 ± 16.8	0.003	145.08 ± 16.2	151.5 ± 17.2	0.503
Δ PR (ms)^**^	13.1 ± 34.7	22.5 ± 32.6	0.206	16.9 ± 38.2	63 ± 74.5	0.496
Maximal Δ PR (ms)^**,&^	37.8 ± 34.6	49.7 ± 32.6	0.052	55.09 ± 43.4	120 ± 27.2	0.003
Δ QRS (ms)	52.2 ± 16.5	53.7 ± 15.9	0.648	47.0 ± 13.8	53.8 ± 18.4	0.755
Maximal Δ QRS (ms)^&^	56.4 ± 16.5	61.09 ± 16.9	0.22	50.7 ± 14.9	62.1 ± 16.5	0.191
Days to LBBB	0	0	> 0.99	1 (1–2.5)	2 (1–3)	0.72
Day of maximal PR duration^**^	0 (0–3)	2 (0–4)	0.19	4 (2–5)	3 (1.5–3)	0.25
Day of maximal QRS duration	0 (0–1)	1 (0–2)	0.2	2 (1–5)	3 (2–3)	0.97
Persistent LBBB upon discharge^	112 (51.8)	30 (96.7)	< 0.001	17 (68)	7 (100)	0.15

LBBB = left bundle branch block; N/L/R = normal/left/right; PPI = permanent pacemaker implantation; TAVR = transcatheter aortic valve replacement

^*^ 2 patients from the immediate and 1 from the delayed LBBB groups, who died on days 0, 1 and 4 post TAVR were excluded from this analysis

^**^ Data not available for 25 patients with atrial fibrillation (20 with immediate LBBB and 5 with delayed LBBB)

^$^ In first ECG documentation of LBBB after TAVR

^#^ Longest documented PR/QRS during hospital follow-up

^&^ Δ between maximal PR/QRS to pre-TAVR PR/QRS

^ Persistent to discharge or to in hospital pacemaker implantation or mortality

A multivariable logistic regression model among patients with early onset LBBB including: valve size, pre TAVR QRS axis (normal vs. abnormal), post TAVR maximal QRS and maximal Δ PR, adjusted for age and sex, demonstrated that pre TAVR QRS axis (OR 4.62, 95% CI 1.775–12.03, *p* = 0.002) and maximal QRS (OR 1.036, 95% CI 1.005–1.068, *p* = 0.022) were independently associated with PPI need at 2-weeks post TAVR; valve size was borderline significant (HR 1.216, 95% CI 0.99–1.487, *p* = 0.057). Of note, due to missing data in a sizeable number of patients, calcium score was not included in the multivariate model.

**Fig. 3:**
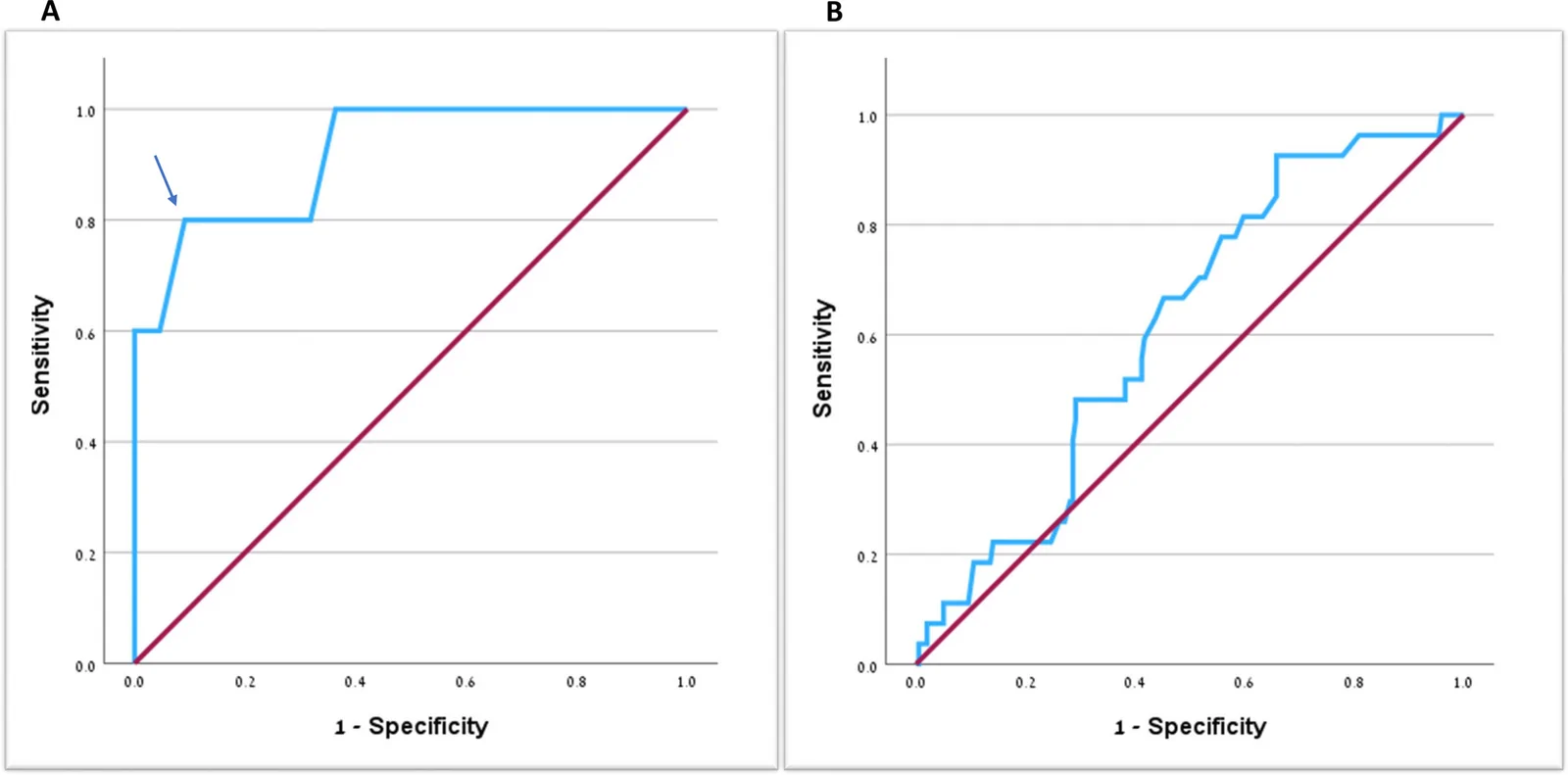
Receiver operating characteristic curve analysis of maximal ΔPR as a predictor of permanent pacemaker implantation among patients with **A**- delayed-onset post TAVR LBBB and **B**- immediate-onset post TAVR LBBB. An area under the curve of 0.92 (95% CI 0.79–1.05, *p* < 0.001) and 0.62 (95% CI 0.52–0.72, *p* = 0.02), in patients with delayed and immediate post TAVR LBBB respectively, was found. The arrow indicates optimal cutoff point for sensitivity and specificity among patients with delayed post-TAVR LBBB (see text)

ROC analysis was conducted to define the best cutoffs of maximal Δ PR and maximal QRS differentiating the need for PPI among patients with delayed and immediate-onset post TAVR LBBB. Regarding Δ PR, an area under the curve of 0.92 (95% CI 0.79–1.05, *p* < 0.001) and 0.62 (95% CI 0.52–0.72, *p* = 0.02), respectively was found (Fig. 3). Among patients with delayed-onset post-TAVR LBBB, a cutoff of maximal Δ PR of 115 ms had a specificity of 91% and a sensitivity of 80% in predicting the need for PPI. While, in patients with immediate post TAVR LBBB, a cutoff of maximal Δ PR ≥ 21 ms had a specificity of 34% and a sensitivity of 93% in predicting a need for PPI.

Regarding maximal QRS, the area under the curve was not statistically significant among patients with delayed onset LBBB (0.59; 95% CI 0.34–0.83, *p* = 0.48). However, in patients with immediate onset LBBB it was 0.67 (95% CI 0.57–0.76, *p* = 0.001) and a cutoff of maximal QRS ≥ 149 ms had a specificity of 49% and a sensitivity of 81% in predicting the need for PPI.

## Discussion

### Main findings

Our study which was conducted on a cohort of 1469 consecutive TAVR patients exhibited, in accordance with previous reports [1–3, 14], that post-TAVR new onset LBBB occurs in ~ 20% of patients. However, it demonstrated that in 11.7% of patients with new-onset LBBB, appearance of LBBB may be delayed and not manifest immediately post TAVR. The clinical consequences of delayed compared to immediate post-TAVR LBBB were shown for the first time, to be comparable, with similar rates of PPI need and mortality, both early and during long term follow-up. Nevertheless, different ECG abnormalities correlated with a need for PPI, while in patients with immediate post TAVR LBBB, baseline abnormal QRS axis and QRS width (mainly ≥ 149 ms) [3] were the dominant discriminating factors, in patients with delayed post TAVR LBBB, the maximal Δ PR differentiated between patients with and without need for PPI. In the delayed post TAVR LBBB group, a cutoff of Δ PR of 115 ms had a sensitivity and specificity of 80% and 91%, respectively for need of PPI.

### Previous studies

Most studies on post TAVR new onset LBBB focused on either, the best approach to risk stratify these patients [1–3, 15], or on LBBB persistence in relation to clinical endpoints, including PPI need, LV function deterioration, and mortality [2, 6, 7, 10, 14]. Few of these studies showed that the appearance of post TAVR new onset LBBB may be delayed in 6.5–15% of patients (Table 4) [6, 10, 16, 17]. Massoullie et al. studied a cohort of 183 patients with post TAVR new onset LBBB [16]. In ~ 15% the appearance of LBBB was delayed (median 2 days, range 1–3 days post TAVR). Urena et al. studied, 61 cases of new-onset post-TAVR LBBB, using balloon- expandable valve. Fifty-seven (93.4%) new-onset LBBB cases were seen immediately on the first post-TAVR ECG, however, in 4 (6.5%), the LBBB appearance was not immediate [6]. Of note, in only 1 of these 4 cases, no conduction abnormality was seen on the first post-TAVR ECG. The other 3 had either, new left anterior hemiblock or incomplete LBBB on their first ECG before developing LBBB. Houthuizen et al. reported an incidence of 12% (21 cases out of 175 new onset post TAVR LBBB) of subacute LBBB, defined as occurring more than 24 h post TAVR [10]. Finally, Testa et al. reported an incidence of 10.1% delayed LBBB (40 out of 394 new onset LBBB cases), based on immediate and predischarge post TAVR ECGs [17]. Rates of LBBB persistence to discharge in patients with immediate versus delayed onset LBBB, according to the above mentioned stuied [6, 10, 16, 17] are presented in Table 4. None of these studies defined the specific characteristics, risk factors for PPI need and prognosis of the delayed post TAVR LBBB patient population.

**Table 4:** Summary of previous studies

	TAVR patients (n)	TAVR type	new onset LBBB	Delayed LBBB cases	Persistence of LBBB to discharge according to immediate vs. delayed onset
Massoullie et al. #16	3228	Mix of BEV/BEV	393 (12.1%), of whom 183 studied	27 (14.7%)	Unknown
Urena et al. #6	202	All BEV	61 (30.2%)	4 (6.5%)	Unknown
Houthuizen et al. #10	476	Mix of BEV/BEV	175 (36.8%)	21^ (12%)	Immediate onset 65%, delayed onset 43%
Testa et al. #17	818*	All SEV	394 (48.2%)	40 (10.1%)	Immediate onset 52%, delayed onset 100%**
Current study	1469	Mix of BEV/BEV	296 (20.1), of whom 282 studied	33 (11.7%)	Immediate onset 57%, delayed onset 72.7%

BEV = balloon-expandable valve; LBBB = left bundle branch block; SEV = self-expandable valve; TAVR = transcatheter aortic valve replacement

^ 21 patients had subacute new onset LBBB defined as occurring more than 24 h post TAVR

^*^Patients without pre TAVR pacemaker or bundle branch block or undergoing permanent pacemaker implantation within 48 h post TAVR

^**^ Based on predischarge ECG, thus, possibly not identifying transient delayed LBBB cases

### Possible mechanism of delayed onset LBBB

TAVR related insult to the conduction system may occur as a consequence of direct trauma during different stages of the procedure, causing immediately manifested conduction abnormality [1, 8]. Post procedurally, continued mechanical pressure, valve self-expansion and development of edema inflammation and fibrosis may further compromise the conduction system [1] manifesting as further worsening of an immediately manifested conduction abnormality or development of new delayed conduction abnormality. The finding that valve size inversely correlates with the delayed appearance of LBBB is consistent with the aforementioned description. Implantation of a larger valve may lead to immediate trauma to the conduction system, whereas a smaller valve may negatively impact the conduction system through more delayed mechanisms, as outlined above. Of note, in the current study, prolonged baseline PR has been associated with the delayed onset of LBBB. The rationale behind this association has not yet been explored, and further validation is necessary.

### ECG parameters associated with PPI need in immediate versus delayed post-TAVR LBBB and clinical implications.

In patients with immediate onset LBBB, in accordance with prior publications, QRS width (especially ≥ 149 ms) and baseline abnormal axis were independently associated with PPI need [3]. Other non-ECG related factors including valve size and calcium score (which was not included in the multivariate analysis), were borderline significantly associated with the need for PPI only in the immediate LBBB group. Whether this possible associations reflects that direct compression by the implanted valve, particularly in the presence of severe valvular calcification, represents a primary mechanism of severe conduction system impairment in patients with immediate-onset LBBB, needs further confirmation.

Interestingly, in the current study, patients with delayed onset LBBB could be stratified into high and low likelihood of PPI need within 2 weeks post TAVR, using maximal Δ PR which was mostly > 115 ms in those who underwent PPI. It is possible that insult to different parts along the conduction system accounts for the appearance of delayed LBBB with or without continued conduction worsening and PPI need. In those with delayed LBBB and no significant PR change the insult may be confined to the LBB while in those with significant PR prolongation the insult may involve the His bundle and fibers that are destined to form the LBB or involve both bundles with more pronounced damage to the LBB and thus manifests with both LBBB and PR prolongation with increased risk for developing AV block. The finding that maximal Δ PR was longer in patients with delayed compared to immediate LBBB, regardless of PPI (Table 2), suggests that as a group the average insult to the conduction system was more proximal manifesting with both PR prolongation and LBBB.

Clinically, the current study suggests that patients with delayed LBBB and especially those with significant PR interval prolongation should be monitored for longer times, with extended hospitalization and possibly even monitored post discharge. In addition, EPS, which was seldomly performed in the current cohort, maybe be used as per treating physician practice [15, 16].

Notably, while we have previously published that PR prolongation per se in patients with narrow QRS, is not predictive of the need for PPI [4], its combination with LBBB and especially delayed LBBB may warrant further attention and prolonged monitoring.

## Limitations

An inherent study limitation is related to its observational retrospective design. ECGs were performed daily and were meticulously analyzed. However, continuous telemetry data, both during and post TAVR, was not available for analysis and therefore we cannot rule out the occurrence of few transient LBBB or AVB cases that went unnoticed. Despite analyzing a large database of almost 1500 TAVR cases, the ultimate number of patients with delayed LBBB and especially those who needed PPI was relatively small, precluding performance of multivariate analysis, and enabling type II error due to small numbers (notably, there was a nonsignificant trend to worse outcomes in the delayed LBBB group). Further larger studies may clarify this point. Finally, according to the study design, clinical, procedural and ECG findings that differ between groups were note, however, the cause of delayed versus immediate post TAVR LBBB was not explored. Nevertheless, our main goal was to describe the phenomenon of delayed LBBB and possible risk factors for PPI once it occurred.

## Conclusion

Around 12% of post-TAVR new onset LBBB cases are delayed and not seen immediately after the procedure. Prognosis of these patients, in terms of PPI need and mortality, is not significantly different from patients with immediate post TAVR-LBBB. However, ECG parameters that correspond to higher PPI risk are different. While QRS duration and baseline abnormal QRS axis correlates with PPI need in patients with immediate LBBB, Δ PR > 115 ms correlates with PPI need in patients with delayed LBBB.

## Supplementary Information

Below is the link to the electronic supplementary material.

Supplementary file1 (DOCX 29 KB)

Supplementary file2 (PPTX 355 KB)

## Data Availability

The data underlying this article will be shared on reasonable request to the corresponding author.

## Abbreviations

AVB: Atrio-ventricular block
ECG: Electrocardiogram
EPS: Electrophysiological study
LBBB: Left bundle branch block
PPI: Permanent pacemaker implantation
RBBB: Right bundle branch block
ROC: Receiver operator characteristics
SAVR: Surgical aortic valve replacement
SSS: Sick sinus syndrome
TAVR: Transcatheter aortic valve replacement
