# Supplementary material for: Immediate vs delayed left bundle branch block after transcatheter aortic valve replacement: incidence and risk factors for permanent pacemaker implantation
Source: J Interv Card Electrophysiol. 2026 Feb 19;69(7):1859–69. doi: 10.1007/s10840-026-02248-3 (PMC13687371; doi:10.1007/s10840-026-02248-3)
Supplement: Supplementary file 1 [file 10840_2026_2248_MOESM1_ESM.docx]

**Supplementary Table 1: Electrophysiological study results.**

| Delayed LBBB | Days to LBBB | Days to EPS | Maximal PR | Maximal QRS | Maximal Δ PR | Maximal Δ QRS | AH | HV | 2-weeks PPI |
| --- | --- | --- | --- | --- | --- | --- | --- | --- | --- |
| 1 | 1 | 5 | 302 | 177 | 124 | 77 | 250 | 49 | no |
| 2 | 1 | 5 | 289 | 161 | 109 | 61 | 165 | 73 | no |
| 3 | 1 | 11 | 250 | 136 | 64 | 28 | 183 | 58 | no |
| 4 | 3 | 6 | 236 | 138 |  | 52 | 180 | 90 | yes |
| 5 | 3 | 6 | 310 | 151 | 114 | 51 | 200 | 52 | no |
| 6 | 0 | 5 | 253 | 167 | 83 | 70 | 130 | 55 | no |
|  |  |  |  |  |  |  |  |  |  |
|  |  |  |  |  |  |  |  |  |  |
| Immediate LBBB Day to LBBB | | Days to EPS | Maximal PR | Maximal QRS | Maximal Δ PR | Maximal Δ QRS | AH | HV | 2-weeks PPI |
| 1 | 0 | 5 | 320 | 155 | 40 | 67 |  | 68 | no |
| 2 | 0 | 5 | 221 | 159 | 13 | 49 | 114 | 50 | no |
| 3 | 0 | 3 | 312 | 142 | 72 | 68 | 315 | 52 | no |
| 4 | 0 | 4 | 276 | 150 | 80 | 56 | 170 | 76 | no |
| 5 | 0 | 7 | 208 | 178 | 66 | 63 |  | 54 | no |
| 6 | 0 | 6 | 200 | 173 | 60 | 83 |  | 77 | yes |
| 7 | 0 | 6 | 217 | 154 | 67 | 74 | 110 | 70 | no |
| 8 | 0 | 13 | 281 | 158 | 67 | 62 | 116 | 67 | no |
| 9 | 0 | 4 | 293 | 185 | 123 | 105 | 220 | 67 | no |
| 10 | 0 | 10 | 292 | 142 | 34 | 24 | 176 | 60 | no |
| 11 | 0 | 2 | 179 | 165 | 9 | 65 | 92 | 48 | no |
| 12 | 0 | 4 | 340 | 162 | 90 | 76 | 202 | 65 | no |
| 13 | 0 | 2 | 180 | 172 | -2 | 57 | 101 | 46 | no |
| 14 | 0 | 3 | 243 | 162 | 27 | 72 | 130 | 67 | no |
| 15 | 0 | 5 | 320 | 183 | 54 | 95 |  | 83 | yes |
| 16 | 0 | 6 | 230 | 146 | 28 | 40 | 150 | 60 | no |
| 17 | 0 | 4 | 231 | 161 | 11 | 46 | 146 | 67 | no |
| 18 | 0 | 4 | 300 | 197 | 60 | 84 | 236 | 52 | no |
| 19 | 0 | 4 | 223 | 154 | 86 | 60 | 145 | 50 | no |
| 20 | 0 | 5 | 313 | 185 | 73 | 75 | 190 | 60 | no |
| 21 | 0 | 7 | 221 | 157 | 21 | 73 |  | 80 | yes |
| 22 | 0 | 2 | 238 | 163 | 78 | 57 | 187 | 59 | no |
| 23 | 0 | 4 | 209 | 154 | 25 | 64 | 56 | 69 | no |
| 24 | 0 | 6 | 204 | 156 | 54 | 56 |  | 80 | yes |
| 25 | 0 | 3 | 224 | 168 | 28 | 64 | 150 | 65 | no |
| 26 | 0 | 5 | 215 | 178 | 45 | 78 | 144 | 65 | no |
| 27 | 0 | 5 | 261 | 156 | 81 | 64 | 145 | 60 | no |
| 28 | 0 | 6 | 180 | 150 | 27 | 62 | 100 | 65 | no |
| 29 | 0 | 2 | 258 | 171 | 84 | 54 | 144 | 59 | no |

All data in milliseconds. AH- atrio-His; EPS= Electrophysiological study; HV=His-ventricular.

Maximal ΔPR is missing in 1 patient from the delayed LBBB group due to atrial fibrillation on baseline ECG

AH is missing in 6 patients from the immediate LBBB group.

**Supplementary Table 2. Clinical characteristics of patients with immediate and delayed LBBB with and without early PPI within 2 weeks**

|  | **Immediate LBBB** | | **p Value** | **Delayed LBBB** | | **p Value** |
| --- | --- | --- | --- | --- | --- | --- |
|  | **(n=247) *** | |  | **(n=32) *** | |  |
|  | **No PPI (n=216)** | **PPI (n=31)** |  | **No PPI (n=25)** | **PPI (n=7)** |  |
| Age (years) | 81.01±6.6 | 78.03±10.3 | 0.229 | 81.29±7.7 | 79.28±9.5 | 0.642 |
| Female sex (%) | 109 (50.5) | 15 (48.4) | 0.829 | 16 (64.0) | 1(14.3) | 0.033 |
| BMI (Kg/m^2^) | 27.8±5.2 | 27.1±4.8 | 0.538 | 29.7±4.8 | 30.2±5.1 | 0.774 |
| Diabetes Mellitus (%) | 95 (44.0) | 8 (25.8) | 0.055 | 10 (40.0) | 4 (57.1) | 0.669 |
| Hypertension (%) | 190 (88.0) | 22 (71.0) | 0.023 | 21 (84.0) | 7 (100.0) | 0.552 |
| Hyperlipidemia (%) | 158 (73.1) | 19 (61.3) | 0.171 | 20 (80.0) | 6 (85.7) | >0.99 |
| Chronic Renal Failure (%) | 48 (22.2) | 6 (19.4) | 0.718 | 3 (12.0) | 1 (14.3) | >0.99 |
| Atrial Fibrillation/Flutter (%) | 56 (25.9) | 9 (29.0) | 0.713 | 10 (40.0) | 3 (42.9) | >0.99 |
| Prior IHD (%) | 114 (52.8) | 13 (41.9) | 0.259 | 10 (40.0) | 5 (71.4) | 0.209 |
| Beta-blockers (%) | 129 (59.7) | 12 (38.7) | 0.027 | 18 (72.0) | 4 (57.1) | 0.648 |
| ACE inhibitors/ARB (%) | 136 (63.0) | 13 (41.9) | 0.025 | 13 (52.0) | 4 (57.1) | >0.99 |
| Amiodarone (%) | 28 (13.0) | 3 (9.7) | 0.776 | 3 (12.0) | 1 (14.3) | >0.99 |
| LVEF (%) | 55.6±7.9 | 55.6±7.7 | 0.882 | 56.8±6.4 | 57.8±5.6 | 0.755 |
| Aortic valve area (cm^2^) | 0.63±0.14 | 0.63±0.19 | 0.736 | 0.59±0.13 | 0.63±0.18 | 0.656 |
| Peak gradient (mmHg) | 80.5±24.6 | 86.5±27.9 | 0.079 | 80.2±25.2 | 74.5±13.06 | 0.688 |
| Mean gradient (mmHg) | 47.5±15.4 | 52±15.6 | 0.068 | 48±16.1 | 44.1±9.1 | 0.562 |
| Calcium score (HU) ^ | 2406±1461 | 3358±2363 | 0.083 | 2440.7±1525 | 2490±1396 | 0.923 |
| **Procedural characteristics** |  |  |  |  |  |  |
| Trans femoral access | 215 (99.5) | 30 (96.8) | 0.236 | 25 (100.0) | 7 (100.0) |  |
| Valve mechanism (BEV/SEV) | 95 (44)/121 (56) | 11 (35.5)/20 (64.5) | 0.371 | 10 (40)/15(60) | 6 (85.7)/1 (14.3) | 0.083 |
| Valve size | 26.2±2.3 | 27.6±2.5 | 0.007 | 27.6±3.04 | 26.9±2.8 | 0.688 |
| Transient CAVB during TAVR | 10 (4.6) | 4 (12.9) | 0.082 | 1 (4.0) | 0 (0.0) | >0.99 |
| Transient QRS widening during TAVR | 0 (0) | 0 (0) | >0.99 | 6 (24) | 2 (28.6) | >0.99 |
| Temporary pacemaker | 158 (73) | 27 (87) | 0.12 | 4 (16) | 6 (86) | 0.001 |
| **In hospital post procedure events/ management** | |  |  |  |  |  |
| EPS | 25 (11.6) | 4 (12.9) | 0.77 | 5 (20) | 1 (14.3) | >0.99 |
| CAVB or HDAVB during hospitalization | 0 (0) | 22 (70.1) | <0.001 | 0 (0) | 5 (71.4) | <0.001 |
| Hospitalization time (days), median (IQR) | 4.5 (3-6) | 7 (3-8) | 0.49 | 6 (3-11) | 7 (6-8) | 0.47 |

AADs= anti arrhythmic drugs; ARB = angiotensin II receptor blocker; ACE = angiotensin converting enzyme; BEV = balloon-expandable valve; BMI = body mass index; CAVB = complete atrioventricular block; EPS = electrophysiological study; HDAVB = high-degree atrioventricular block; IHD = heart ischemic disease; LVEF = left ventricular ejection fraction, PPI = permanent pacemaker implantation; SEV = self-expandable valve; TAVR = Transcatheter aortic valve replacement.

^*^ 2 patients from the immediate and 1 from the delayed LBBB groups, who died on days 0, 1 and 4 post TAVR (before 2 weeks post TAVR) were excluded from this analysis. ^Data not available for 75 and 8 patients from the immediate and delayed LBBB subgroups, respectively.
